# Strain Specific Factors Control Effector Gene Silencing in *Phytophthora sojae*

**DOI:** 10.1371/journal.pone.0150530

**Published:** 2016-03-01

**Authors:** Sirjana Devi Shrestha, Patrick Chapman, Yun Zhang, Mark Gijzen

**Affiliations:** 1 Agriculture and Agri-Food Canada, 1391 Sandford Street, London, Ontario, Canada; 2 Department of Biology, University of Western Ontario, London, Ontario, Canada; USDA ARS, UNITED STATES

## Abstract

The *Phytophthora sojae* avirulence gene *Avr3a* encodes an effector that is capable of triggering immunity on soybean plants carrying the resistance gene *Rps3a*. *P*. *sojae* strains that express *Avr3a* are avirulent to *Rps3a* plants, while strains that do not are virulent. To study the inheritance of *Avr3a* expression and virulence towards *Rps3a*, genetic crosses and self-fertilizations were performed. A cross between *P*. *sojae* strains ACR10 X P7076 causes transgenerational gene silencing of *Avr3a* allele, and this effect is meiotically stable up to the F_5_ generation. However, test-crosses of F_1_ progeny (ACR10 X P7076) with strain P6497 result in the release of silencing of *Avr3a*. Expression of *Avr3a* in the progeny is variable and correlates with the phenotypic penetrance of the avirulence trait. The F_1_ progeny from a direct cross of P6497 X ACR10 segregate for inheritance for *Avr3a* expression, a result that could not be explained by parental imprinting or heterozygosity. Analysis of small RNA arising from the *Avr3a* gene sequence in the parental strains and hybrid progeny suggests that the presence of small RNA is necessary but not sufficient for gene silencing. Overall, we conclude that inheritance of the *Avr3a* gene silenced phenotype relies on factors that are variable among *P*. *sojae* strains.

## Introduction

The genus *Phytophthora* includes some 120 species of plant pathogenic organisms [1,2]. Root and stem rot of soybean caused by *Phytophthora sojae* is a destructive, soil-borne plant disease that has spread globally since its identification in North America in the 1950s [3,4]. *Phytophthora sojae* is a diploid, homothallic organism. Self-fertilized oospores can develop from one strain, while out-crossing between strains results in F_1_ hybrids. It is not possible to distinguish hybrid progeny from self-fertilized progeny without the use of strain-specific markers. The first experimental hybridizations of *P*. *sojae* were identified by crossing strains differing in drug resistance, but now DNA markers are routinely used for tracking parentage [5–10]. Many different traits and markers have been followed in F_1_ and F_2_ progeny. Segregation patterns usually follow Mendelian rules but loss of heterozygosity in F_1_ or F_2_ progeny commonly occurs in *P*. *sojae*, and in other *Phytophthora* species, and can result in unusual inheritance patterns [9,11–15].

The disease caused by *P*. *sojae* is managed through the selection, breeding, and deployment of soybean cultivars with enhanced resistance. This process relies on naturally occurring resistance (*Rps*) genes conferring race-specific immunity to *P*. *sojae*, and on quantitative trait loci (QTL) that condition partial resistance [16–19]. Variation of *P*. *sojae* makes management difficult, because it can evolve rapidly into new strains that defeat *Rps* gene mediated immunity. Pathogen effectors that trigger immunity in the host are avirulence (Avr) factors. Changes to *Avr* genes that result in escape from host immunity cause gain of virulence.

The *P*. *sojae Avr* genes identified to date include *Avr1a* [20], *Avr1b* [21], *Avr1c* [22], *Avr1d* [23,24], *Avr1k* [25], *Avr3a/5* [26], *Avr3b* [27], *Avr3c* [28], and *Avr4/6* [29]. Strain specific gain of virulence changes are diverse but transcriptional polymorphisms are common, having been observed for *Avr1a*, *Avr1b*, *Avr1c*, *and Avr3a/5*. Loss of effector gene mRNA may be due to conventional mutations that disable transcription but emerging results suggest that epigenetic changes can also underlie these polymorphisms [30–32].

The *Avr3a/5* locus of *P*. *sojae* was identified through genetic mapping and transcriptional profiling [20,26]. Two independent soybean genes conditioning resistance to *P*. *sojae*, *Rps3a* and *Rps5*, differentially recognize allelic variants at the *Avr3a/5* locus. Escape from *Rps3a* mediated immunity is caused by transcriptional variation. Transcripts of *Avr3a* mRNA are present in avirulent strains and progeny but not in the virulent strains and progeny [26]. Crossing of *P*. *sojae* strain P6497 (avirulent to *Rps3a*) and strain P7064 (virulent to *Rps3a*) and analysis of F_1_ and F_2_ progeny demonstrated that expression of *Avr3a* and avirulence to *Rps3a* segregate normally as dominant Mendelian traits in this cross [20]. The *P*. *sojae* strain P7064 harbors insertion and deletion mutations in the promoter of *Avr3a* that likely interfere with transcription and cause it to segregate as a recessive allele. In contrast, crosses between *P*. *sojae* strain P7076 (avirulent to *Rps3a*) and strain ACR10 (virulent to *Rps3a*) result in transgenerational gene silencing of *Avr3a* and gain of virulence to *Rps3a* in all F_1_ and F_2_ progeny, despite that the *Avr3a* alleles themselves segregate normally [30]. This past study additionally showed that small RNA (sRNA) molecules arising from the *Avr3a* gene are abundant in gene silenced parental strains and progeny, but are absent or reduced in those that express the gene.

Experiments described here further explore the phenomenon of naturally occurring gene silencing of *Avr3a* in its inheritance in *P*. *sojae*. We test the meiotic stability of gene silencing of *Avr3a* over multiple generations and determine whether gene silenced alleles of *Avr3a* gain the capability to silence expressed alleles of other strains. We also construct a new cross of *P*. *sojae* strains ACR10 X P6497 and follow the inheritance of virulence to *Rps3a*, the expression of *Avr3a*, and the presence of sRNA, in parental strains and hybrid progeny. Overall, our results indicate that strain-specific epistatic factors play a role in controlling the expression of *Avr3a* in hybrid progeny.

## Results

### Gene silencing of *Avr3a* is meiotically stable over multiple generations in progeny from a cross of ACR10 X P7076

To study the meiotic stability of gene silencing, F_4_ progeny were developed by self-fertilization of four different F_3_ progeny. The four different F_3_ (F_3_-40, F_3_-60, F_3_-83, and F_3_-87) isolates were selected randomly from progeny with the genotype *Avr3a*^*P7076*^*/Avr3a*^*P7076*^, and self-fertilized to produce F_4_ progeny. From each of the four F_3_ progeny, four germinating oospores were isolated to develop F_4_ progeny. Therefore a total of 16 F_4_ progeny were generated. Virulence assays were performed on test (*Rps3a*) and control (*rps3a*) soybean plants, and the cultures were assessed for the presence of *Avr3a* mRNA transcript by reverse transcriptase polymerase chain reaction (RT-PCR). The virulence assays show that 15/16 of the progeny are virulent towards soybeans carrying the *Rps3a* gene, but one F_4_ lost general virulence or pathogenicity, being avirulent to both test (*Rps3a*) and control (*rps3a*) plants (Table 1). There are no detectable *Avr3a* mRNA transcripts among all (16/16) tested F_4_ progeny including the avirulent individual.

**Table 1:** Virulence outcomes and *Avr3a* transcript detection in F_3_ and F_4_ progeny from *P*. *sojae* cross ACR10 X P7076.

F_3_ progeny^1^	F_4_ progeny^1^	Virulence on *Rps3a* (L83-570)^2^	Virulence on *rps3a* (Williams)^2^	Assigned phenotype^3^	*Avr3a* mRNA transcript (RT-PCR)^4^
F_3_-40		60	60	V	-
	F_4_-40(3)	9	15	NP	-
	F_4_-40(4)	43	45	V	-
	F_4_-40(5)	60	60	V	-
	F_4_-40(7)	60	60	V	-
F_3_-60		60	60	V	-
	F_4_-60(1)	60	60	V	-
	F_4_-60(2)	55	60	V	-
	F_4_-60(4)	53	56	V	-
	F_4_-60(5)	60	60	V	-
F_3_-87		58	60	V	-
	F_4_-87(1)	60	60	V	-
	F_4_-87(2)	55	53	V	-
	F_4_-87(3)	60	60	V	-
	F_4_-87(4)	60	60	V	-
F_3_-83		60	60	V	-
	F_4_-83(1)	60	60	V	-
	F_4_-83(2)	60	60	V	-
	F_4_-83(3)	60	60	V	-
	F_4_-83(4)	59	60	V	-

^1^All progeny originally derived from F_2_ individuals homozygous for the *Avr3a*^*P7076*^ allele

^2^ Number of dead plants from a total of 60 challenged, representing the sum from two independent virulence assays of 30 plants each.

^3^ Phenotypes: A, avirulent; V, virulent; NP, non-pathogenic.

^4^ Symbol (+) positive for *Avr3a* mRNA, (-) negative for *Avr3a* mRNA. All samples were positive for control *Actin* mRNA. Summary of results from two independent biological replicates.

Similarly, to develop F_5_ progeny, one individual from each of the four different F_4_ strains was selected and processed for self-fertilization. The isolate that had lost virulence to control plants was excluded, but otherwise the four individuals were selected randomly. From each F_4_ self-fertilization, four oospores were isolated and developed into F_5_ progeny, thus making a total of 16 F_5_ progeny. Virulence assays were performed on test (*Rps3a*) and control (*rps3a*) soybean plants, and the cultures were tested for the presence of *Avr3a* transcript by RT-PCR. The virulence assay results show that 15/16 of the F_5_ progeny are virulent towards soybeans carrying the *Rps3a* gene, but again one F_5_ lost general virulence or pathogenicity, since it is avirulent to both test (*Rps3a*) and control (*rps3a*) plants (Table 2). There are no detectable *Avr3a* mRNA transcripts among all (16/16) tested F_5_ progeny. Although one isolate in each F_4_ and F_5_ progeny lost their general virulence towards soybean plants with or without *Rps3a*, it was not due to changes in expression of the *Avr3a* transcript. Therefore, *Avr3a* gene silencing was stable in all F_4_ and F_5_ progeny.

**Table 2:** Virulence outcomes and *Avr3a* transcript detection in F_4_ and F_5_ progeny from *P*. *sojae* cross ACR10 X P7076.

F_4_ progeny^1^	F_5_ progeny^1^	Virulence on *Rps3a* (L83-570)^2^	Virulence on *rps3a* (Williams)^2^	Assigned phenotype^3^	*Avr3a* mRNA transcript (RT-PCR)^4^
F_4_-40(7)		60	60	V	-
	F_5_-40(1)	20	12	NP	-
	F_5_-40(2)	59	57	V	-
	F_5_-40(3)	60	59	V	-
	F_5_-40(4)	60	60	V	-
F_4_-60(5)		60	60	V	-
	F_5_-60(1)	60	60	V	-
	F_5_-60(2)	53	52	V	-
	F_5_-60(3)	55	56	V	-
	F_5_-60(4)	54	47	V	-
F_4_-87(1)		60	60	V	-
	F_5_-87(1)	60	60	V	-
	F_5_-87(2)	56	58	V	-
	F_5_-87(3)	60	60	V	-
	F_5_-87(4)	45	46	V	-
F_4_-83(3)		60	60	V	-
	F_5_-83(1)	60	60	V	-
	F_5_-83(2)	60	60	V	-
	F_5_-83(3)	60	60	V	-
	F_5_-83(4)	60	60	V	-

^1^All progeny originally derived from F_2_ individuals homozygous for the *Avr3a*^*P7076*^ allele

^2^ Number of dead plants from a total of 60 challenged, representing the sum from two independent virulence assays of 30 plants each.

^3^ Phenotypes: A, avirulent; V, virulent; NP, non-pathogenic.

^4^ Symbol (+) positive for *Avr3a* mRNA, (-) negative for *Avr3a* mRNA. All samples were positive for control *Actin* mRNA. Summary of results from two independent biological replicates.

### Gene silencing of *Avr3a* is released by out-crossing

**Fig 1:**
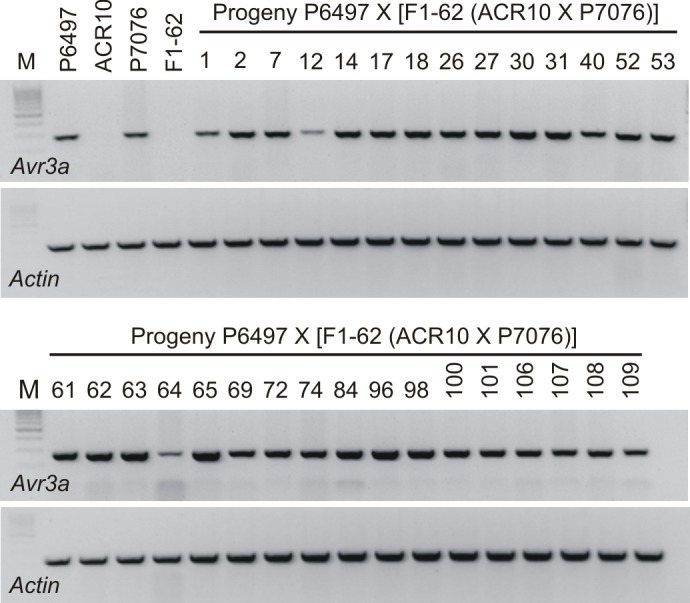
Analysis for *Avr3a* transcripts in parental *P*. *sojae* strains and progeny of test cross P6497 X [F_1_-62(ACR10 X P7076)]. The DNA products from RT-PCR analysis are shown after electrophoresis in agarose gels and visualization with fluorescent dye. The cDNA was synthesized using total RNA isolated from the mycelia of *P*. *sojae*. RT-PCR was carried out using gene specific primers for the *P*. *sojae Avr3a* and *Actin* genes. *P*. *sojae* strains are indicated above each lane; M, marker lane.

To determine whether silenced alleles of *Avr3a*^*P7076*^ have the capacity to silence expressed alleles of other strains of *P*. *sojae*, a test cross was performed between *P*. *sojae* strains P6497 X [F_1_(ACR10 X P7076)]. A total of 110 oospores were isolated from a cross of P6497 X [F_1_-62(ACR10 X P7076)]. Due to the identical *Avr3a* sequence of P6497 and ACR10, hybrids of interest (*Avr3a*^*P6497*^/*Avr3a*^*P7076*^ or *Avra*^*P6497*^/*Avr3a*^*ACR10*^) were identified using three different co-dominant DNA markers. A total of 14 *Avr3a*^*P6497*^/*Avr3a*^*P7076*^ and 17 *Avr3a*^*P6497*^/*Avr3a*^*ACR10*^ hybrids were identified from the 110 progeny of this cross. Virulence testing of the 31 hybrids indicated that 30 are avirulent towards test (*Rps3a*) plants, whereas one (progeny number 12; *Avr3a*^*P6497*^/*Avr3a*^*ACR10*^) is virulent (Table 3). Most (30/31) isolates retained virulence towards control (*rps3a*) plants. Analysis of *Avr3a* mRNA levels by RT-PCR showed that all 31 cultures produce *Avr3a* transcripts. However, the *Avr3a* mRNA level is reduced in progeny number 12 that is virulent on *Rps3a* plants (Fig 1).

**Table 3:** Genotypes, virulence outcomes and *Avr3a* transcript detection in test cross progeny of P6497 X F_1_-62 (ACR10 X P7076).

Test cross progeny number and *Avr3a* genotype	Virulence on *Rps3a*(L83-570)^1^	Virulence on *rps3a* (Williams)^1^	Assigned phenotype^2^	*Avr3a* mRNA transcript(RT-PCR)^3^
1 (*Avr3a*^*P6497*^*/Avr3a*^*ACR10*^)	0	60	A	+
2 (*Avr3a*^*P6497*^*/Avr3a*^*P7076*^)	0	60	A	+
7 (*Avr3a*^*P6497*^*/Avr3a*^*ACR10*^)	0	60	A	+
12 (*Avr3a*^*P6497*^*/Avr3a*^*ACR10*^)	60	60	V	+
14 (*Avr3a*^*P6497*^*/Avr3a*^*P7076*^)	1	60	A	+
17 (*Avr3a*^*P6497*^*/Avr3a*^*ACR10*^)	0	60	A	+
18 (*Avr3a*^*P6497*^*/Avr3a*^*ACR10*^)	0	60	A	+
27 (*Avr3a*^*P6497*^*/Avr3a*^*P7076*^)	2	60	A	+
30 (*Avr3a*^*P6497*^*/Avr3a*^*ACR10*^)	0	60	A	+
31 (*Avr3a*^*P6497*^*/Avr3a*^*P7076*^)	0	60	A	+
40 (*Avr3a*^*P6497*^*/Avr3a*^*ACR10*^)	0	60	A	+
51 (*Avr3a*^*P6497*^*/Avr3a*^*ACR10*^)	0	60	A	+
52 (*Avr3a*^*P6497*^*/Avr3a*^*ACR10*^)	0	58	A	+
53 (*Avr3a*^*P6497*^*/Avr3a*^*ACR10*^)	0	60	A	+
61 (*Avr3a*^*P6497*^*/Avr3a*^*P7076*^)	0	0	NP	+
62 (*Avr3a*^*P6497*^*/Avr3a*^*ACR10*^)	0	60	A	+
63 (*Avr3a*^*P6497*^*/Avr3a*^*P7076*^)	0	55	A	+
64 (*Avr3a*^*P6497*^*/Avr3a*^*ACR10*^)	0	56	A	+
65 (*Avr3a*^*P6497*^*/Avr3a*^*ACR10*^)	0	60	A	+
69 (*Avr3a*^*P6497*^*/Avr3a*^*P7076*^)	0	60	A	+
72 (*Avr3a*^*P6497*^*/Avr3a*^*ACR10*^)	0	60	A	+
74 (*Avr3a*^*P6497*^*/Avr3a*^*P7076*^)	0	60	A	+
84 (*Avr3a*^*P6497*^*/Avr3a*^*P7076*^)	0	59	A	+
96 (*Avr3a*^*P6497*^*/Avr3a*^*ACR10*^)	0	60	A	+
98 (*Avr3a*^*P6497*^*/Avr3a*^*P7076*^)	0	60	A	+
100 (*Avr3a*^*P6497*^*/Avr3a*^*P7076*^)	0	60	A	+
101 (*Avr3a*^*P6497*^*/Avr3a*^*ACR10*^)	0	60	A	+
106 (*Avr3a*^*P6497*^*/Avr3a*^*P7076*^)	0	60	A	+
107 (*Avr3a*^*P6497*^*/Avr3a*^*P7076*^)	0	60	A	+
108 (*Avr3a*^*P6497*^*/Avr3a*^*ACR10*^)	0	60	A	+
109 (*Avr3a*^*P6497*^*/Avr3a*^*P7076*^)	0	60	A	+

^1^ Number of dead plants from a total of 60 challenged, representing the sum from two independent virulence assays of 30 plants each.

^2^ Phenotypes: A, avirulent; V, virulent; NP, non-pathogenic.

^3^ Symbol (+) positive for *Avr3a* mRNA, (-) negative for *Avr3a* mRNA. All samples were positive for control *Actin* mRNA. Summary of results from two independent biological replicates.

**Fig 2:**
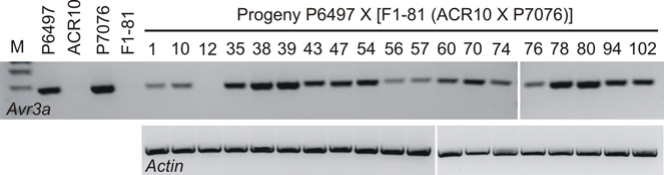
Analysis for *Avr3a* transcripts in parental *P*. *sojae* strains and progeny of test cross P6497 X [F_1_-81(ACR10 X P7076)]. The DNA products from RT-PCR analysis are shown after electrophoresis in agarose gels and visualization with fluorescent dye. The cDNA was synthesized using total RNA isolated from the mycelia of *P*. *sojae*. RT-PCR was carried out using gene specific primers for the *P*. *sojae Avr3a* and *Actin* genes. Data is missing in this analysis for *Actin* control of parental strains and F_1_-81; however, *Avr3a* expression results for these samples are consistent with previous analyses that included controls. *P*. *sojae* strains are indicated above each lane; M, marker lane.

**Fig 3:**
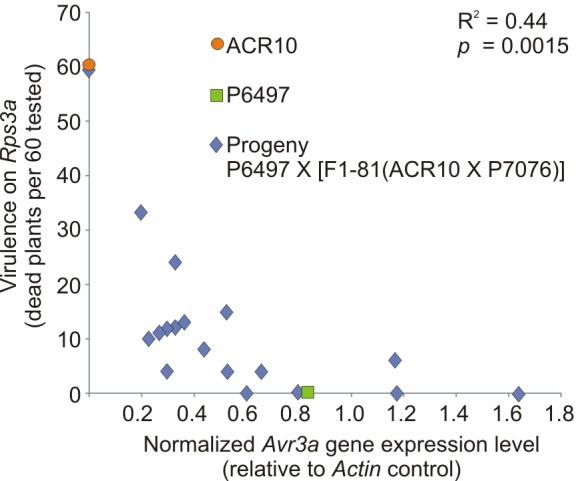
Quantitative expression analysis of *Avr3a* and virulence towards *Rps3a*, among the progeny from test cross of *P*. *sojae* strains P6497 X [F_1_-81(ACR10 X P7076)]. Expression of *Avr3a* was determined by quantitative real-time PCR analysis. Virulence towards *Rps3a* was determined by a hypocotyl inoculation bioassay. Expression values are a mean, and virulence numbers are a sum of plants killed, derived from two independent biological replicates. Expression of *Avr3a* was normalized with *P*. *sojae Actin*. Parental *P*. *sojae* strains P6497 and ACR10 are included for comparison. The correlation (R^2^) and probability (*p*) from a regression analysis is also shown.

This experiment was replicated using another F_1_ individual. The cross of *P*. *sojae* P6497 X [F_1_-81(ACR10 X P7076)] was performed and a total of 19 hybrids of interest were identified; 6/19 were *Avr3a*^*P6497*^/*Avr3a*^*ACR10*^and 13/19 were *Avr3a*^*P6497*^/*Avr3a*^*P7076*^. Analysis for *Avr3a* transcripts by RT-PCR showed that 18/19 of the hybrids possess *Avr3a* mRNA, whereas it is not detectable in the remaining (virulent) culture (Table 4). Among the 18 individuals with detectable *Avr3a* transcript, the signal intensity of *Avr3a* amplification product appears to be variable (Fig 2). Likewise, from the virulence assays it was found that the phenotypic penetrance of the avirulence trait in several isolates was incomplete because not all plants with *Rps3a* gene were killed (Table 4). To more accurately measure the *Avr3a* gene expression level among the cultures, quantitative real-time PCR was performed. Comparison of the results from the quantitative real-time PCR analysis and virulence tests demonstrates a correlation, with higher expression levels of *Avr3a* leading to greater phenotypic penetrance of the avirulence trait (Fig 3).

**Table 4:** Genotypes, virulence outcomes and *Avr3a* transcript detection in test cross progeny of P6497 X F_1_-81(ACR10 X P7076).

Test cross progeny number and *Avr3a* genotype	Virulence on *Rps3a* (L83-570)^1^	Virulence on *rps3a* (Williams)^1^	Fisher’s test *p*-value^2^	Assigned phenotype^3^	*Avr3a* mRNA transcript (RT-PCR)^4^
1 *(Avr3a*^*P6497*^*/Avr3a*^*P7076*^*)*	33	60	1.7E-10	A’	+
10 *(Avr3a*^*P6497*^*/Avr3a*^*P7076*^*)*	24	60	6.6E-15	A’	+
12 *(Avr3a*^*P6497*^*/Avr3a*^*P7076*^*)*	60	60	1	V	-
35 *(Avr3a*^*P6497*^*/Avr3a*^*P7076*^*)*	4	60	6.6E-30	A	+
38 *(Avr3a*^*P6497*^*/Avr3a*^*ACR10*^*)*	6	60	9.4E-28	A	+
39 *(Avr3a*^*P6497*^*/Avr3a*^*ACR10*^*)*	5	60	8.5E-29	A	+
43 *(Avr3a*^*P6497*^*/Avr3a*^*ACR10*^*)*	12	60	1.6E-22	A’	+
47 *(Avr3a*^*P6497*^*/Avr3a*^*P7076*^*)*	2	60	2E-32	A	+
54 *(Avr3a*^*P6497*^*/Avr3a*^*P7076*^*)*	8	60	7.7E-26	A	+
56 *(Avr3a*^*P6497*^*/Avr3a*^*ACR10*^*)*	10	60	4.1E-24	A’	+
57 *(Avr3a*^*P6497*^*/Avr3a*^*P7076*^*)*	11	60	2.7E-23	A’	+
60 *(Avr3a*^*P6497*^*/Avr3a*^*P7076*^*)*	13	60	8.9E-22	A’	+
70 *(Avr3a*^*P6497*^*/Avr3a*^*ACR10*^*)*	3	60	4.1E-31	A	+
74 *(Avr3a*^*P6497*^*/Avr3a*^*P7076*^*)*	4	60	6.6E-30	A	+
76 *(Avr3a*^*P6497*^*/Avr3a*^*ACR10*^*)*	8	60	7.7E-26	A	+
78 *(Avr3a*^*P6497*^*/Avr3a*^*P7076*^*)*	0	60	1E-35	A	+
80 *(Avr3a*^*P6497*^*/Avr3a*^*P7076*^*)*	0	60	1E-35	A	+
94 *(Avr3a*^*P6497*^*/Avr3a*^*P7076*^*)*	0	60	1E-35	A	+
102 *(Avr3a*^*P6497*^*/Avr3a*^*P7076*^*)*	9	60	5.9E-25	A	+

^1^ Number of dead plants from a total of 60 challenged, representing the sum from two independent virulence assays of 30 plants each.

^2^ Probability value from Fisher’s exact test, on whether the kill rate on test plants (*Rps3a*) differs from the kill rate on control plants (*rps3a*).

^3^ Phenotypes: A, avirulent; V, virulent; A’, incomplete avirulent.

^4^ Symbol (+) positive for *Avr3a* mRNA, (-) negative for *Avr3a* mRNA. All samples were positive for control *Actin* mRNA. Summary of results from two independent biological replicates.

**Fig 4:**
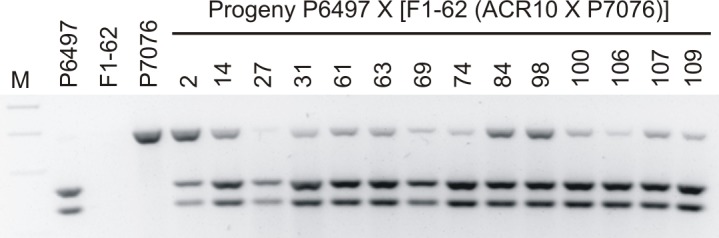
Analysis for allele specific expression of *Avr3a* in *P*. *sojae* progeny from the test cross P6497 X [F_1_-62(ACR10 X P7076)]. The DNA products from RT-PCR analysis are shown after restriction enzyme digestion, electrophoresis and visualization with fluorescent dye. Purified mRNA transcripts from *P*. *sojae* parental strains and test cross progeny with the genotype *Avr3a*^*P6497*^/*Avr3a*^*P7076*^ were analyzed to determine allele specific expression. The RT-PCR was performed using *Avr3a* specific primers and the amplified product was digested with the restriction enzyme *AluI*. The *Avr3a*^*P6497*^allele contains a restriction site for *AluI* whereas the *Avr3a*^*P7076*^ allele does not. *P*. *sojae* strains are indicated above each lane; M, marker lane.

In order to determine allele specific expression of *Avr3a* in progeny from the test cross P6497 X [F1-62(ACR10 X P7076)], RT-PCR was performed on mRNA samples, and reaction products were digested with *Alu*I. The *Avr3a*^*P6497*^allele contains a restriction site for *AluI* but the *Avr3a*^*P7076*^ allele does not [26]. In this cross, an allele-specific expression test is only feasible for progeny with the genotype *Avr3a*^*P6497*^/*Avr3a*^*P7076*^. It is not possible to determine whether there is allele-specific expression for progeny with the genotype *Avr3a*^*P6497*^/*Avr3a*^*ACR10*^ since the two alleles are sequence identical. Results from this analysis show that the test cross progeny with the genotype *Avr3a*^*P6497*^/*Avr3a*^*P7076*^ produce transcripts of both alleles; thus the silenced *Avr3a* allele of P7076 was released when test crossed with P6497 (Fig 4).

### Cross of *P*. *sojae* strains P6497 X ACR10 results in F_1_ hybrids that segregate for virulence towards *Rps3a*

The *P*. *sojae* strains P6497 and ACR10 possess sequence identical alleles of *Avr3a* but differ in expression; P6497 expresses the gene whereas ACR10 does not. The results from the test crosses of P6497 X [F_1_ (ACR10 X P7076)] (described above) indicate that the *Avr3a*^*ACR10*^ allele itself does not have the capability to silence the *Avr3a*^*P6497*^ allele, although the actual expression levels of *Avr3a* were variable among the progeny. To further test the interaction between the *Avr3a*^*P6497*^ and *Avr3a*^*ACR10*^ alleles, we performed a direct cross between *P*. *sojae* strains P6497 X ACR10.

**Fig 5:**
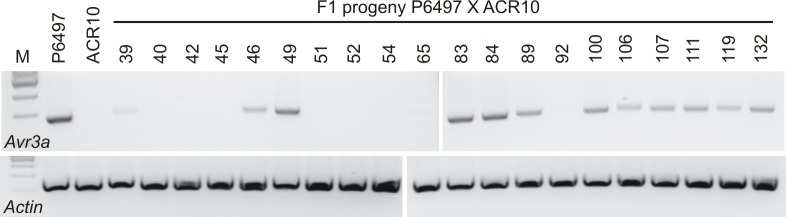
Analysis for *Avr3a* transcripts in parental *P*. *sojae* strains and progeny of cross P6497 X ACR10. The DNA products from RT-PCR analysis are shown after electrophoresis in agarose gels and visualization with fluorescent dye. The cDNA was synthesized using total RNA isolated from the mycelia of *P*. *sojae*. RT-PCR was carried out using gene specific primers for the *P*. *sojae Avr3a* and *Actin* genes. *P*. *sojae* strains are indicated above each lane; M, marker lane.

From a total of 220 oospores, 20 F_1_ hybrids were identified; the remaining 200 offspring resulted from self-fertilization of parental strains P6497 and ACR10. Results from the RT-PCR analysis of *Avr3a* mRNA demonstrate that the transcript is detectable in 12/20 F_1_ progeny whereas it is not detectable in 8/20. Plant inoculation assays show that all hybrid progeny lacking *Avr3a* transcripts are virulent towards *Rps3a*. Most progeny with detectable *Avr3a* transcripts are avirulent towards *Rps3a* but phenotypic penetrance of this trait was incomplete in some individuals, likely due to variability of expression of the *Avr3a* gene as estimated by the RT-PCR analysis (Fig 5, Table 5).

**Table 5:** Parentage, virulence outcomes, and *Avr3a* transcript detection in F_1_ hybrids of *P*. *sojae* P6497 X ACR10

F_1_ progeny	Maternal parent	Virulence on *Rps3a* (L83-570)^1^	Virulence on *rps3a* (Williams)^1^	Fisher’s test *p*-value^2^	Assigned phenotype^3^	*Avr3a* mRNA transcript (RT-PCR)^4^
39	P6497	59	60	0.5	V	+
40	ACR10	60	60	1	V	-
42	ACR10	60	60	1	V	-
45	P6497	56	60	0.05936	V	-
46	P6497	4	60	6.6E-30	A	+
49	P6497	18	53	2.8E-11	A’	+
51	ACR10	59	60	0.5	V	-
52	ACR10	54	56	0.21033	V	-
54	ACR10	45	60	1.1E-05	V’	-
65	P6497	60	60	1	V	-
83	ACR10	17	60	5.1E-19	A’	+
84	P6497	0	60	1E-35	A	+
89	ACR10	12	60	1.6E-22	A’	+
92	P6497	60	60	1	V	-
100	P6497	39	60	5.7E-08	A’	+
106	P6497	0	60	1E-35	A	+
107	P6497	2	60	2E-32	A	+
111	ACR10	7	60	9E-27	A	+
119	P6497	0	60	1E-35	A	+
132	ACR10	5	60	8.5E-29	A	+

^1^ Number of dead plants from a total of 60 challenged, representing the sum from two independent virulence assays of 30 plants each.

^2^ Probability value from Fisher’s exact test, on whether the kill rate on test plants (*Rps3a*) differs from the kill rate on control plants (*rps3a*).

^3^ Phenotypes: A, avirulent; V, virulent; A’, incomplete avirulent; V’, incomplete virulent.

^4^ Symbol (+) positive for *Avr3a* mRNA, (-) negative for *Avr3a* mRNA. All samples were positive for control *Actin* mRNA. Summary of results from two independent biological replicates.

### Maternal or paternal effects do not determine gene silencing of *Avr3a* in F_1_ progeny from P6497 X ACR10

**Fig 6:**
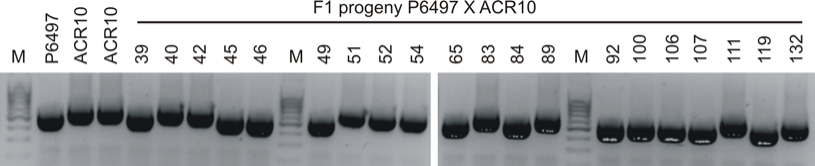
Mitochondrial DNA marker analyses of parental strains and F_1_ hybrids from a cross of *P*. *sojae* strains P6497 X ACR10. The DNA products from PCR analysis are shown after electrophoresis in agarose gels and visualization with fluorescent dye. Shown are PCR amplified products of a mitochondrial DNA fragment from *P*. *sojae* strains P6497 and ACR10, and their F_1_ progeny; M, marker lane.

The apparent 1:1 segregation of *Avr3a* expression in the F_1_ hybrids from P6497 X ACR10 suggests a parental effect, such as imprinting or heterozygosity, might be influencing the outcome. Therefore we identified the maternal parent of hybrids using a mitochondrial DNA marker (Fig 6, Table 5). For the 20 F_1_ progeny from the P6497 X ACR10 cross, results show that P6497 is the maternal parent for 11/20, and of these, 8/11 are positive for *Avr3a* expression. The ACR10 strain is the maternal parent for 9/20, and of these, 4/9 are positive for *Avr3a* expression. Therefore, the results do not correlate and there are no apparent maternal or paternal effects on *Avr3a* gene silencing for progeny from this cross.

Parental heterozygosity at a locus controlling *Avr3a* expression or silencing is another possible explanation for 1:1 segregation ratios in F_1_ progeny. To determine whether potential heterozygosity within the parental strains P6497 and ACR10 could account for the variation in virulence and *Avr3a* gene expression in the F_1_ progeny, a total of 50 self-fertilized oospores were isolated from each of the strains. Out of total 50 progeny developed by self-fertilization of strain ACR10, all (50/50) lack detectable *Avr3a* transcripts by RT-PCR analysis. Most of the progeny (45/50) are virulent towards test (*Rps3a*) and control (*rps3a*) plants, but some (5/50) are intermediate in virulence towards test (*Rps3a)* and/or control (*rps3a)* soybean plants (S1 Table). All of the self-fertilized progeny (50/50) developed by P6497 X P6497 are avirulent towards soybean *Rps3a* and possess detectable *Avr3a* transcripts (S2 Table). Results indicate that the apparent 1:1 segregation of *Avr3a* expression in the P6497 X ACR10 progeny is unlikely to be due to parental heterozygosity, since each of the parental strains were true breeding for *Avr3a* expression.

### Segregation of virulence towards *Rps3a*, and *Avr3a* expression in F_2_ progeny from P6497 X ACR10

To study the segregation pattern of the *Avr3a* virulence trait and gene expression in F_2_ progeny from the cross P6497 X ACR10, independent F_2_ populations were created by self-fertilization of six different F_1_ individuals. Three F_2_ populations were from virulent F_1_s lacking *Avr3a* mRNA transcripts, and three were from avirulent F_1_s that express the *Avr3a* gene. A total of 150 F_2_ progeny were isolated, including 75 for each of the two classes of F_1_s. Results show that all 75 F_2_ individuals arising from self-fertilization of virulent F_1_s lacking *Avr3a* transcripts were virulent towards soybean carrying *Rps3a* gene, and do not possess *Avr3a* mRNA transcripts (Table 6). By contrast, the F_2_ populations from avirulent F_1_ progeny segregate for *Avr3a* expression and virulence towards *Rps3a*. The segregation of virulent:avirulent phenotypes matches a 1:3 ratio, by chi-squared analysis.

**Table 6:** Segregation analysis for F_2_ populations developed from virulent and avirulent F_1_ hybrids of P6497 X ACR10.

Phenotype^1^	F_1_	F_2_	F_2_ Phenotype^2^		*p-*value^3^
		Population *n*	Avirulent mRNA(+) *n*	Virulent mRNA(-) *n*	
F_1_ hybrids silenced for *Avr3a* and virulent towards *Rps3a*					
	F_1_-51	25	0	25	-
	F_1_-52	25	0	25	-
	F_1_-92	25	0	25	-
	Total	75	0	75	-
F_1_ hybrids expressing *Avr3a* and avirulent towards *Rps3a*					
	F_1_-46	25	19	6	0.91
	F_1_-111	25	21	4	0.30
	F_1_-119	25	16	9	0.20
	Total	75	56	19	0.95

^1^ Selected F_1_ hybrids from P6497 X ACR10

^2^ Virulence towards *Rps3a* and presence of *Avr3a* mRNA. F_2_ progeny with avirulent phenotype were positive for *Avr3a* mRNA transcript (+) and progeny with virulent phenotype were negative for *Avr3a* mRNA transcript (-), without exception, as determined by RT-PCR.

^3^ *p*-value from χ^2^ analysis for 3:1 segregation

### The presence of *Avr3a* small RNA molecules is usually associated with silencing in parental strains and progeny

**Fig 7:**
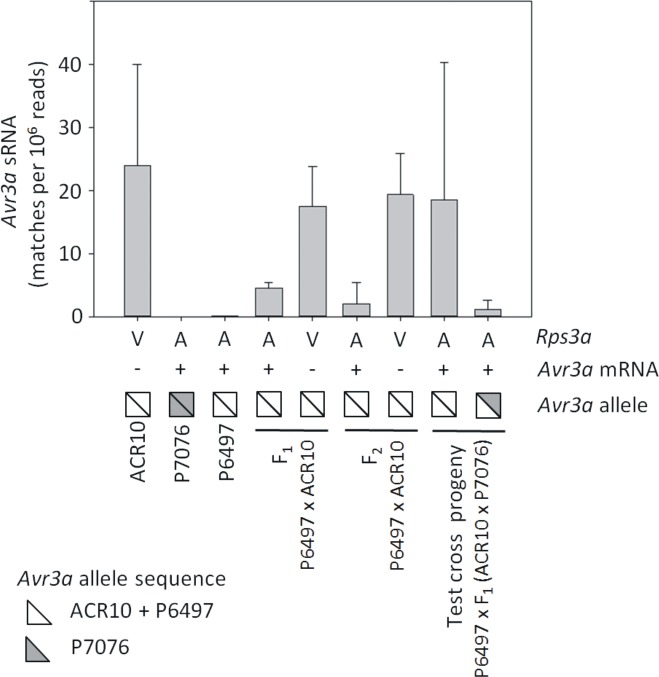
Prevalence of small RNA to *Avr3a* in parental strains and hybrids of *P*. *sojae*. Shown are the mean and range of values for matches of sRNA to *Avr3a*, as determined by deep sequencing of sRNA libraries. Values are from three independent biological replicates, with the exception of strain P7076 for which there were two replicates. Below the graph is shown the virulence of to *Rps3a* (V, virulent; A, avirulent); the presence (+) or absence (-) of *Avr3a* mRNA transcripts as determined by RT-PCR; the DNA sequence genotype at the *Avr3a* locus; and the *P*. *sojae* strain or progeny identification. *P*. *sojae* strains P6497 and ACR10 possess sequence identical alleles of *Avr3a* but differ in expression.

We previously showed that *Avr3a* sRNA of 25 nt in length are abundant in the gene silenced strain ACR10 but not in the expressing strain P7076. All progeny from the cross ACR10 X P7076 were gene silenced for *Avr3a* and possessed abundant sRNA, regardless of genotype [30]. To measure the levels of *Avr3a* sRNAs progeny from the test cross P6497 X [F_1_(ACR10 X P7076)] and from the direct cross P6497 X ACR10, we performed deep sequencing and counted sRNA matching to *Avr3a*. Nine different *P*. *sojae* strains or hybrids were tested and three biological replicates were performed for each (with one exception, data is available for only two replicates of parental strain P7076), for a total of 26 samples. The results are summarized in Fig 7 and S3 Table. The results indicate that *Avr3a* gene silenced strains or hybrids always possess abundant *Avr3a* sRNA (values ranging from 10 to 42 matches per 10^6^ reads). Gene expressing strains or hybrids usually possessed far less *Avr3a* sRNA, but this result was not consistent among the hybrid progeny. Parental strains expressing *Avr3a* (P7076 and P6497) had the lowest levels of *Avr3a* sRNA (values ranging from 0 to 0.13 matches per 10^6^ reads). Hybrid progeny expressing *Avr3a* often had higher amounts of *Avr3a* sRNA than parental strains with the same phenotype. In fact, the highest amount of *Avr3a* sRNA that we measured (43 matches per 10^6^ reads) was from a test cross progeny that phenotypically expresses *Avr3a* and is avirulent to *Rps3a*. However, this was an exception, and all of the other hybrid progeny expressing the gene possessed lower levels of sRNA (values ranging from 0 to 6.3 matches per 10^6^ reads). Overall, the results suggest that *Avr3a* sRNA is necessary but not sufficient for gene silencing.

## Discussion

Recent research has indicated that the variation of *Avr3a* mRNA transcript levels determines the virulence of *P*. *sojae* towards soybean plants carrying the resistance gene *Rps3a*. Strains that possess *Avr3a* mRNA transcripts are avirulent but strains lacking *Avr3a* mRNA transcripts are virulent towards soybean plants carrying *Rps3a* [20,26]. It has also been found that crosses between *P*. *sojae* strains P7076 (avirulent phenotype; *Avr3a*^*P7076*^*/ Avr3a*^*P7076*^) X ACR10 (virulent phenotype; *Avr3a*^*ACR10*^*/ Avr3a*^*ACR10*^) causes transgenerational gene silencing of *Avr3a* and gain of virulence in all F_1_ and F_2_ progeny, despite normal segregation of the *Avr3a* gene itself [30]. Our results now demonstrate that gene silencing of *Avr3a* is meiotically stable in progeny from *P*. *sojae* strains P7076 X ACR10 to the F_5_ generation. No *Avr3a* mRNA transcripts were detected in any of the progeny. The virulence assay results indicate that all the progeny retained virulence towards soybean cultivars carrying the *Rps3a* gene, with the exception of one outlier isolate in each of F_4_ and F_5_ progeny that suffers from a general loss of pathogenicity. The loss of pathogenicity, indicated by a loss of virulence towards control plants lacking any known *Rps* genes, was also observed at low frequency among the self-fertilized ACR10 progeny. We cannot account for the spontaneous loss of pathogenicity of these cultures, but it is a phenomenon that has long been known to occur in *P*. *sojae* [18,33].

The stability of *Avr3a* silencing was tested because it is known from other examples of transgenerational or inherited epigenetic phenomena that gene silencing can fade over generations or be influenced by environmental effects [34–37]. This could occur if an epigenetic factor required for silencing is not fully self-propagating or is (de)activated by an environmental condition. Meiosis is also a process that can re-set epigenetic control, especially for developmental or environmentally induced epigenetic changes that occur during somatic cell division [38,39]. Results from our experiments demonstrate that *Avr3a* gene silencing in progeny from *P*. *sojae* ACR10 X P7076 is stable through F_4_ and F_5_ progeny. Additionally, since the original cross was made well before the current experiments were conducted, the results show that *Avr3a* silencing is temporally stable over at least five years. Thus, if an inherited epigenetic factor is responsible for *Avr3a* silencing in this cross, then this factor is exceptionally stable under the experimental conditions tested here.

Another reason to test stability of *Avr3a* gene silencing was to determine whether *Avr3a* expression could be reconstituted in F_4_ or F_5_ progeny. If transgenerational effects are simply the result of a multiple independent loci segregating in the cross, that are necessary for *Avr3a* expression but that are not recovered in the proper combination in the F_2_ progeny, then reconstitution of *Avr3a* expression could occur in further generations. In simple epistatic silencing events, one would expect to find recombination and segregation in F_2_ populations, and reconstitution of transcription of the *Avr3a* gene within some or many of the F_2_ progeny. This was not observed. Results show that reconstitution of *Avr3a* expression did not occur in any of the F_4_ or F_5_ progeny, so it is not possible to conclude with certainty whether multiple epistatic loci are required. Nonetheless, a parsimonious interpretation of the outcome tends to discount the multiple-epistasis hypothesis. The scenario that *P*. *sojae* strain ACR10 is lacking multiple independent factors, each required in a homozygous condition specifically for *Avr3a* gene expression, in a combination that was never recovered in any of the progeny, is possible but implausible.

Other hypotheses invoking epistatic loci to account for the silencing of *Avr3a* in progeny from *P*. *sojae* ACR10 X P7076 are possible, such as chromosomal changes, high frequency gene conversion, or loss of heterozygosity. These phenomena can occur in hybrid cultures generated from crossing different strains, and have been demonstrated to varying degrees in a number of oomycete species including *P*. *sojae*, *P*. *infestans*, *P*. *parasitica*, *P*. *cinnamomi* and *Pythium ultimum*, [9,14,40–42]. For example, if *P*. *sojae* strains ACR10 and P7076 differ at an epistatic locus that is necessary for *Avr3a* gene expression or silencing, then gene conversion, loss of heterozygosity or chromosomal changes affecting the epistatic locus in hybrid cultures could cause unusual inheritance patterns. However, this scenario requires special assumptions. First, the hypothetical epistatic factor must exclusively convert to the haplotype which results in *Avr3a* silencing. Second, this change must occur instantaneously in all hybrid progeny. Although there are only a limited number of studies of gene conversion and loss of heterozygosity in *P*. *sojae*, the results tend to contrast with the observations of *Avr3a* inheritance in the ACR10 X P7076 cross. Loss of heterozygosity may show strong allele-specific bias but it is unusual for all hybrid progeny to exclusively convert to one allele [9]. Models and previous observations also suggest that loss of heterozygosity is time dependent, and tends to accumulate in hybrid cultures as they are clonally propagated, rather than occurring instantaneously [9]. Therefore, the characteristics of *Avr3a* gene silencing in hybrid progeny differ from the known examples of loss of heterozygosity in *P*. *sojae*, but it is not possible to discount this hypothesis as an explanation.

One of the goals of this study was to determine whether the silenced alleles of *Avr3a* have an ability to silence the expressed alleles of other *P*. *sojae* strains, as was previously shown in the cross of *P*. *sojae* strains ACR10 X P7076. The outcome from the test cross of P6497 X [F_1_(ACR10 X P7076)] demonstrates that all progeny with the genotype *Avr3a*^*P6497*^*/Avr3a*^*P7076*^ or *Avr3a*^*P6497*^*/ Avr3a*^*ACR10*^ express *Avr3a* mRNA transcripts. For the progeny with the genotype *Avr3a*^*P6497*^*/Avr3a*^*P7076*^, expression of both alleles was detected, and thus the silencing of the *Avr3a*^*P7076*^ allele was released by out-crossing. For the progeny with the genotype *Avr3a*^*P6497*^*/Avr3a*^*ACR10*^, it was not possible to determine allele specific expression, but it is clear that the *Avr3a*^*ACR10*^ allele does not have the capability to silence the *Avr3a*^*P6497*^ allele in this circumstance. The results differ from classical examples of paramutation, which is a phenomenon of gene-silencing where a paramutagenic allele has the ability to silence the paramutable allele, and paramutable alleles gain the ability to be paramutagenic [43]. However, the known examples of paramutation are diverse in their characteristics, so it remains possible that paramutation and gene silencing of *Avr3a* share underlying mechanistic features.

In the second replicate of the test cross, the phenotypic penetrance of the *Avr3a* avirulence trait was found to be incomplete in many of the progeny. We observed plants with the *Rps3a* gene being killed by inoculation with the progenies from the test cross, despite that expression of *Avr3a* was detected in these cultures. Furthermore, the *Avr3a* transcript level varied among the progeny and correlated well with the virulence profiles of the isolates. The results indicate that expression of the *Avr3a* gene is not fully restored in all of the progeny, and demonstrate a quantitative effect of *Avr3a* expression and avirulence towards the *Rps3a* resistance gene.

It is not known what causes the variation of *Avr3a* transcript levels in the test cross progeny, but the results clearly show that silencing is released by out-crossing and that transgenerational inheritance does not occur. A possible explanation for this outcome is involvement of strain specific factors in *Avr3a* gene expression and/or silencing. As previously discussed, a hypothetical epistatic factor necessary for *Avr3a* expression or silencing can be invoked to explain the unusual inheritance patterns in progeny from the ACR10 X P7076 cross, if one assumes all the special conditions are met. Likewise, the release of gene silencing in the test cross hybrids could be due to the presence of strain specific factors in *P*. *sojae* P6497 that regulate expression of *Avr3a* or that control epigenetic inheritance.

The results from the P6497 X ACR10 cross show both differences and similarities to the results from the test cross of P6497 X [F_1_(ACR10 X P7076)]. The test cross results were different because all 23 progeny with the heterozygous genotype *Avr3a*^*P6497*^*/Avr3a*^*ACR10*^ were avirulent towards soybean *Rps3a* and expressed *Avr3a* transcripts, whereas these traits segregated in F_1_ from the direct cross of P6497 X ACR10 that had the identical heterozygous genotype *Avr3a*^*P6497*^*/Avr3a*^*ACR10*^. Nonetheless, for the F_1_ progeny that produced detectable *Avr3a* mRNA, from either cross, the expression level appeared to be variable, and this influenced the phenotypic penetrance of the avirulence trait.

The apparent 1:1 segregation of *Avr3a* expression in the F_1_s from P6497 X ACR10 could not be explained by parent-of-origin effects or by heterozygosity of the parental strains. To further study the inheritance of *Avr3a* expression in progeny from the P6497 X ACR10 outcross, we developed F_2_ populations from virulent and avirulent F_1_ hybrids. The F_2_ populations developed from virulent F_1_ individuals were all virulent and silenced for *Avr3a* expression, whereas these traits segregated in F_2_s derived from avirulent F_1_ individuals. The overall segregation ratio of *Avr3a* expressing: *Avr3a* silenced F_2_ progeny was 56:19, which fits a 3:1 ratio. These results suggest that *Avr3a* expression is inherited as a simple dominant trait in the F_2_ progeny that develop from F_1_ hybrids expressing *Avr3a*.

A possible explanation to account for the results from the P6497 X ACR10 cross is that there is a factor necessary for the expression of *Avr3a* present in *P*. *sojae* strain P6497 but not in ACR10, such as a factor that suppresses the silencing of *Avr3a*. In half the F_1_ hybrid progeny, this factor is somehow lost, resulting in the presence of *Avr3a*-silenced F_1_ individuals that are true breeding. In F_1_ progeny that express *Avr3a* transcripts, the factor remains in an apparent heterozygous state, so that the F_2_ progeny segregate in a 3:1 ratio for *Avr3a* expression.

Although there clearly must be multiple factors that are necessary for *Avr3a* expression or silencing, invoking gene conversion or loss of heterozygosity of these factors to account for the inheritance of *Avr3a* expression is troublesome because special conditions must be assumed to fully explain the results. There is also evidence from other studies indicating that loss of heterozygosity cannot account for changes in *Avr* gene expression states in *P*. *sojae*. For example, the *P*. *sojae Avr1a* and *Avr1c* genes show apparent epiallelic variation in expression [22]. Sequence identical alleles of each, *Avr1a* and *Avr1c*, can produce mRNA transcripts or be silenced, in a strain-specific manner. The inheritance of gene silencing has not been studied for *Avr1a* or *Avr1c*, but it is known that clonally propagated strains can spontaneously switch expression states for each of these genes [20,22]. Loss of heterozygosity is not likely to underlie the *Avr1a* or *Avr1c* gene expression states in these studies because no hybridization events were involved. It has also been demonstrated that virulence towards *Rps1a* can be repeatedly lost and recovered in successive single zoospore isolates of *P*. *sojae* [44].These observations cannot be explained by gene conversion or loss of heterozygosity, which are irreversible processes [9].

The relationship between gene silencing of *Avr3a* and the presence of corresponding sRNA molecules was investigated by deep sequencing. Our interpretation of the results is that *Avr3a* sRNA are necessary but not sufficient for silencing of the gene. In other organisms where gene silencing mechanisms have been deeply investigated, models indicate that sRNAs must form a complex with protein effectors in order for silencing to be accomplished, whether it is transcriptional or post-transcriptional. Thus, sRNA does not act independently to achieve silencing. One possibility to account for our results is that there is strain specific variation in *P*. *sojae* for components of the silencing complex that enable the uncoupling of sRNA from the *Avr3a* gene silenced phenotype.

The results presented here cannot be easily explained but they do indicate that there is strain-specific interplay between conventional and epigenetic variations in *P*. *sojae*. Support for this conclusion is provided by comparative genomic studies of *Phytophthora infestans* and its sister species demonstrating that epigenetic regulators, such as genes predicted to encode histone methyltransferases, can be highly polymorphic and show signs of positive selection [45]. The modulation of gene expression states by heterochromatin based mechanisms, and by sRNA or transposon sequences, has also been described or proposed to occur in *P*. *infestans* [46–50]. Our results discount a role for parental imprinting of *Avr3a* and help to provide a path forward for discovery of the additional factors that regulate the expression of this gene.

## Materials and Methods

### *Phytophthora sojae* strains

Cultures of *P*. *sojae* parental strains P6497, ACR10, and P7076 were from our collection, Agriculture and Agri-Food Canada, London, ON. No specific permission was required for research on this organism. The origin of the parental strains used in this study has been described [15,20]. Progeny from the cross of ACR10 X P7076 have previously been described [30]. For short term (up to one year) storage, cultures were maintained on 2.5% (v/v) vegetable juice (V8) agar medium at 16°C in the dark. For prolonged storage, parental strains and hybrid progeny were cryo-preserved in liquid nitrogen.

### Plant materials

Soybean (*Glycine max*) cultivar Williams (*rps*) and the corresponding isoline L83-570 (*Rps3a*) were grown in field plots at Agriculture and Agri-Food Canada, London, ON. No specific permission was required for this location or activity. The experiments did not involve the use of transgenic plants. The field use did not involve endangered or protected species. The seeds were harvested and used for virulence assays, as described below.

### Culture and cross of *P*. *sojae*

*Phytophthora sojae* strains were cultured by transferring 5 mm diameter mycelial plugs cut from the growing edge of a culture onto 26% (v/v) vegetable juice (V8) agar (regular) medium and incubated at 25°C for 7 d in the dark. Crossing media plates [2.5% (v/v) V8 juice agar] supplemented with β-sistosterol (10μg/mL), kanamycin (50 μg/mL), ampicillin (100 μg/mL), and rifampicin (10 μg/mL) were freshly prepared. To cross two different strains or to perform self-fertilization of *P*. *sojae*, fresh culture from a growing edge of regular V8 juice medium (5 mm square) was transferred aseptically on the centre of the crossing plate and incubated at 25°C for 7 d in darkness. After incubation, cultures grown on separate plates were homogenized together and co-cultivated on crossing medium plates at 25°C in darkness for 5 weeks. Cultures were homogenized using a 10 mL sterile syringe with 18-gauge needle. Darkness was maintained using aluminium foil to wrap the plates.

### Generation of F_4_ and F_5_ progeny from *P*. *sojae* cross ACR10 X P7076

Selected F_3_ progeny with the genotype *Avr3a*^*P7076*^/*Avr3a*^*P7076*^ were available from the cross ACR10 X P7076. These cultures were self-fertilized to produce F_4_ and F_5_ progeny. The original ACR10 X P7076 cross was previously performed and the progeny were maintained in the laboratory [30]. A total of four F_3_ individuals (F_3_-40, F_3_-60, F_3_-83, and F_3_-87) were revived from cryo-storage, and self-fertilized to generate F_4_ progeny; similarly, F_5_ progeny were developed from the F_4_ progeny.

### Isolation of oospores

All the steps for isolation of oospores were performed aseptically at a laminar flow bench. Out-crossing or self-fertilization was performed as described above. After maturation, oospores were isolated by maceration, filtration, centrifugation, and other purification steps, as previously outlined [16] and described below. After incubation for 5 weeks, four plates (for each crossing) of matured cultures were sliced using a sterile blade. Using a pre-chilled commercial blender (Waring), the diced material from all four plates was homogenized with 100 mL of 4°C sterile water. Two pulses of one min each were performed, so as not to overheat the sample.

The cultures were then sieved through three sterile 75 μm nylon membrane to remove agar and mycelium. The filtrate was collected into a 50 mL sterile conical tube and frozen at -20°C for 24 hours to kill the hyphae. In the following day, the culture was thawed at 45°C water bath for 10 minutes and re-filtered using three sterile 75 μm nylon membranes. The filtrate was collected into a 50 mL sterile conical tube and centrifuged at 3,000 rpm for 10 minutes. Mycelial fragments and agar which floated on the supernatant were removed using a sterile Pasteur pipette. A darkly coloured pellet of oospores can be observed after centrifugation. This pellet was then washed three times with sterile water.

The pellet containing oospores was treated with β-glucuronidase (2000 units/mL suspension) and incubated at 37°C for 16 h. After incubation, the oospore suspension was centrifuged and washed three times and re-suspended into 20 mL of sterile distilled water. Kanamycin (50 μg/mL) and ampicillin (100 μg/mL) were added to the suspension and oospores were spread on 1.5% (w/v) water agar plates supplemented with 10 μg/mL β-sistosterol and rifampicin (10 μg/mL), approximately 500 oospores per plate (90 x 16 mm). The oospores on the water agar plates were incubated at 25°C in the dark. Oospores begin to germinate after 2–4 days. Plates were checked every other day using a stereomicroscope (60X magnification). Germinating oospores were transferred from the water agar plate to regular V8 juice media using a sterile diamond-head transfer needle and incubated at 25°C for 7 days in the dark. The cultures grown from pure isolated oospores were then used for further DNA and RNA work.

### Genomic DNA isolation

Genomic DNA was extracted from mycelial cultures of *P*. *sojae* strains using standard phenol chloroform extraction followed by isopropanol precipitation. Cultures of *P*. *sojae* arising from single oospores were transferred to regular V8 medium and incubated at 25°C for 7 days. A lawn of *P*. *sojae* mycelia growing on the surface of the V8 juice medium was collected by scraping with a sterile pipette tip (blunt end). The mycelia was then transferred into a 2 mL Eppendorf tube containing 1 mL mycelial extraction buffer and frozen at -20°C for 24 hours. The mycelia extraction buffer contained: 200 mM Tris HCl (pH 8.5), 250 mM NaCl, 25 mM EDTA, 2% SDS, and sterile water. The next day, the frozen sample was thawed at room temperature. Phenol (750 μL) was added, and the sample was vortexed and centrifuged (13,000 rpm for 15 minutes). The supernatant containing the DNA was collected and re-extracted again with phenol. The aqueous suspension was collected, and phenol: chloroform (1:1) was added, and the sample was centrifuged at 13,000 rpm for 15 min. A solution (500 μL) of chloroform: isoamyl alcohol (24:1) was; added to the supernatant, and the sample was centrifuged at 13,000 rpm for 15 min. The supernatant was collected and RNA was removed by adding RNase A (1.5 μL of 10 mg/ml). Isopropanol (60% volume of total DNA suspension) was added to the DNA suspension and the sample was kept at -20°C overnight to precipitate the DNA. The DNA pellet was obtained by centrifugation (13,000 rpm for 30 min at 4°C). The DNA pellet was then washed with 70% (v/v) ethanol, centrifuged at 13,000 rpm for 5 min, and air dried (10 minutes). The dried pellet was dissolved in a solution of 10 mM Tris HCl and 0.1 mM EDTA, heated at 65°C for 10 min and stored at -20°C.

### Polymerase chain reaction (PCR)

For all PCR amplifications, a three step PCR method was applied. Initial denaturation temperature of the PCR reaction was 94°C for 2 min. The three step cycle was: denaturation (94°C for 40 sec), annealing (annealing temperature was according to primers used for 40 sec), and extension (72°C for 1 min). After completion of all cycles, a 72°C extension temperature was applied for 10 min. The number of PCR cycles varied according to the purpose of the experiment; for restriction digestion, 40 cycles were used, for RT-PCR, 30 cycles were used. The PCR amplification was performed using 15 ng genomic DNA as template. The PCR mixture (25 μL) was prepared using following reaction components: 0.2 mM dNTPs, 1.5 mM MgCl_2_, 0.5 μM of each forward and reverse primers, 1.25 U Taq DNA polymerase and 1X PCR buffer as recommended by the supplier (Invitrogen, Life Technologies). Gel electrophoresis was performed to resolve products, which were visualized using a fluorescent dye (SYBR safe, Invitrogen).

### Hybrid determination

To determine the hybrid progeny (P6497/ACR10 or P6497/P7076) from crosses of P6497 X F_1_ (ACR10 X P7076), two different co-dominant cleaved amplified polymorphisms (CAPs) markers were analysed. The *Scaf-29-M2* sequence (448 bp) with strain-specific sequence polymorphisms was amplified using forward primer (5'-CCCTCGAGAACGCCAACTT-3') and reverse primer (5'-CCTCGCTCGCCTTCATCC-3'). A list of all primer sequences is provided in (S4 Table). The *Scaf-29-M2* sequences of strain ACR10 and P7076 possess a restriction site for the enzyme *Eco*RV but strain P6497 does not. Similarly, *Avh320* sequence (400 bp) was amplified using forward primer (5'-AACGCTCTCGAAAGTGGC-3') and reverse primer (5'-AAAGAACTTCGACAG CC-3'). The *Avh320* sequences of strains P6497 and ACR10 have a restriction site for *Cla*I but strain P7076 does not. To track segregation of the *Avr3a*^*P6497*^ and *Avr3a*^*P7076*^ *or Avr3a*^*ACR10*^ alleles, forward primer (5'-GCTGCTTCCTTCCTGGTTGC-3') and reverse primer (5'-GCTGCTGCCTT TTGCTTCTC-3') were used and amplified *Avr3a* sequences were digested with restriction enzyme *Alu*I. The *Avr3a* sequences of ACR10 and P6497 include a restriction site for *Alu*I but the *Avr3a* sequence of P7076 does not.

### Restriction Digestion

To analyse the CAPs markers, PCR amplification was performed as described above using 15 ng genomic DNA as template. For restriction digestion of amplified sequences, 10 μL volumes of amplified products and 10 μL enzyme mixtures (3 U of respective enzyme, buffer and BSA (New England Bio labs) were mixed and incubated according to the optimum temperature of the restriction enzymes. Gel electrophoresis was performed to resolve products, which were visualized using fluorescent stain (SYBR safe, Invitrogen).

### Extraction of RNA and reverse transcriptase polymerase chain reaction (RT-PCR)

To study the expression of the *Avr3a* gene of *P*. *sojae* strains, total RNA was extracted from mycelial tissues. *Phytophthora sojae* isolates were cultured on cellophane (Ultra Clear Cellophane, RPI Crop) placed over 26% (v/v) V8 juice agar medium, and incubated at 25°C for 7 d in darkness. The cellophane disks containing mycelia were then peeled off the agar medium, frozen in liquid nitrogen and stored at -80°C until used. Mycelial tissues were ground in liquid nitrogen to a fine powder. Total RNA was extracted using a solution of phenol-guanidine isothiocyanate (Trizol) according to the instructions provided by the manufacturer (Invitrogen). The steps applied to extract RNA were as follows. In each conical tube containing equal mass of frozen, powdered mycelial tissue, Trizol reagent (4 mL) was added and sample was mixed. The suspension was dispensed into 2 ml Eppendorf tubes and 700 μL chloroform was added. The sample was vortexed, and kept at room temperature for 3 minutes. Tubes were centrifuged at 14,000 rpm at 4°C and the supernatant was collected into 15 mL conical tubes. The supernatant was transferred into 1.5 mL Eppendorf tubes and equal volume of isopropanol was added in each tube. The samples were centrifuged at 14,000 rpm for 30 minutes at 4°C. The supernatant was discarded and pellet was washed with 1 ml 75% ethanol prepared in DEPC treated water, then centrifuged at 14,000 rpm for 3 minutes at 4°C. The RNA pellet in the tube was air dried and dissolved in DEPC treated water and stored at -80°C. Total RNA samples were quantified using a spectrophotometer (NanoDrop, Thermo Scientific, USA) and their integrity was checked by electrophoretic analysis of 200 ng of each sample on 1% (w/v) agarose gel in 1X TAE buffer (40 mM Tris, 40 mM acetate, 1 mM EDTA, pH 8.2–8.4). For RT-PCR, 1 μg of total RNA was treated with DNase I and first-strand cDNA was synthesized using reverse transcriptase (Superscript III, Invitrogen) as per the manufacturer's directions. The *Avr3a* mRNA transcript was detected by PCR with the forward primer (5'-GCTGCTTCC TTCCTGGTT GC-3') and the reverse primer (5'-GCTGCTGCCTTTTGCTTCTC-3'). List of primers used in this project is given in table 2.2. Primers-specific for *P*. *sojae Actin* were used as a control. A minimum of two independent biological replicates, prepared separately and at different times, were performed for each analysis.

### Virulence assay

The standard hypocotyl inoculation based assay was employed to test the virulence of *P*. *sojae* cultures [51]. The soybean cultivars Williams (*rps*) and Williams isoline L83-570 (*Rps3a*) were used. Seeds were sown in 10 cm pots (~15 seeds per pot). Soybean plants were grown for 7 days in a growth chamber which was maintained with 16 h continuous light supply, 25°C day temperature followed by 16°C night temperatures before inoculations. *Phytophthora sojae* cultures were grown on 0.9% (w/v) V8 juice agar medium for 7 d, and macerated using 10 mL syringe with 18-gauge needle. The mycelial slurry (approximately 300 μL) was inoculated in the hypocotyl of soybean plant by making an incision below the epidermal layer. Plants were covered with plastic bags for 3 d, and then left for another 3 d without bags and the disease outcome was scored on day 6.

### Identification of maternal parent in *P*. *sojae* cross P6497 X ACR10

To identify the maternal parent of F_1_ hybrids from the cross P6497 X ACR10, a mitochondrial DNA marker was developed to track the inheritance of this organelle. Polymorphisms in the mitochondrial DNA sequence were identified by comparison of the reference genome P6497 to the re-sequenced strain ACR10, and verified by PCR amplification and Sanger DNA sequencing [52,53]. Specifically, primers were designed to flank an insertion/deletion polymorphism of 96 bp, present in ACR10 but absent from P6497. The PCR reaction was performed using total genomic DNA as template and mitochondrial DNA sequence specific primers (forward primer 5'-TTTGGTGTATAGTTTCCCAACC-3', and reverse primer 5'-CGTGTTACTCACCCGTTCG-3'). The amplicon was then visualized by gel electrophoresis using 2% agarose gel. Maternal parentage of progeny was determined by comparing the size of the amplified product to that of control samples from *P*. *sojae* strains P6497 and ACR10.

### Quantitative real time PCR (qRT-PCR) to study the expression level of *Avr3a* gene among *P*. *sojae* hybrids

To quantitatively measure the expression of *Avr3a* in selected *P*. *sojae* cultures, quantitative real time PCR was performed. To perform quantitative real time PCR, labelled nucleotides (Cyber-Green SuperMix, Quanta Biosciences) were used with real time detection system (CFX96, Bio-Rad Laboratories, Inc., USA). The cDNA was prepared from RNA samples and purified as described above, using reverse transcriptase (Superscript III, Invitrogen). The *Avr3a* gene expression was analysed using gene specific primers (forward primer: 5’-TCGCTCAAGTTGTGG TCGTC-3’ and reverse primer: 5’-TCGACAGCGTCCTATCTTCG-3’). The primers used for reference gene (*Actin*) amplification were forward primer: 5’-CGAAATTGT GCGCG ACATCAAG-3’ and reverse primer: 5’-GGTACCGCCC GACAGCACGAT-3’. The data were analyzed using software (CFX manager, Bio-Rad Laboratories Inc., USA).

### Small RNA sequence analysis

Total RNA was extracted from mycelia cultures of *P*. *sojae* as described above. Construction of small RNA libraries using the Illumina TruSeq small RNA kit and sequencing on an Illumina HiSeq2000 followed instructions provided by the manufacturer. Single reads of 50 nt in length were obtained. Sequences were trimmed by Scythe (https://github.com/vsbuffalo/scythe) and filtered based on Q30 scores. Any sequences less than 21 nt or that Scythe could not trim were discarded. The remaining sequences were compared to a 556 bp target sequence comprised of the *Avr3a* open reading frame (336 bp) and 100 bp of flanking sequence from the adjacent 3’ and 5’ regions, and matching sequences were counted. The number of matches was normalized to the total number of trimmed and filtered sequences for each library. This analysis was performed using *Avr3a* allele sequences from the reference genome strain P6497 and from strain P7076, to determine whether the target allele sequence influenced the number of sRNA matches. The results did not differ substantially between the two analyses.

### Data deposition

Small RNA sequence data for all samples described in this study is available from the National Center for Biotechnology Information Sequence Read Archive, Bioproject PRJNA300858.

## Supporting Information

S1 Table Virulence outcomes and *Avr3a* transcript detection in oospore progeny from self-fertilized *P*. *sojae* strain ACR10. (DOCX)

S2 Table Virulence outcomes and *Avr3a* transcript detection in oospore progeny from self-fertilized *P*. *sojae* strain P6497. (DOCX)

S3 Table Small RNA sequencing of *P*. *sojae* strains and progeny. (DOCX)

S4 Table Oligonucleotide primer sequences. (DOCX)

## References

[1] Erwin DC, Ribeiro OK (1996) Phytophthora Diseases Worldwide. St. Paul, MN: The American Phytopathological Society.

[2] Kroon LP, Brouwer H, de Cock AW, Govers F (2012) The genus phytophthora anno 2012. Phytopathology 102: 348–364. 10.1094/PHYTO-01-11-0025 22185336

[3] Tyler BM, Gijzen M (2014) The *Phytophthora sojae* genome sequence: Foundatoin for a revolution In: Dean RA, Lichens-Park A, Kole C, editors. Genomics of Plant-Associated Fungi and Oomycetes: Dicot Pathogens. Berlin Heidelberg: Springer-Verlag.

[4] Kamoun S, Furzer O, Jones JD, Judelson HS, Ali GS, et al (2015) The Top 10 oomycete pathogens in molecular plant pathology. Mol Plant Pathol 16: 413–434. 10.1111/mpp.12190 25178392 PMC6638381

[5] Layton AC, Kuhn DN (1988) The virulence of interracial heterokaryons of *Phytophthora megasperma* f.sp. *glycinea*. Phytopathology 78: 961–966.

[6] Whisson SC, Drenth A, Maclean DJ, Irwin JA (1994) Evidence for outcrossing in Phytophthora sojae and linkage of a DNA marker to two avirulence genes. Curr Genet 27: 77–82. 7750150 10.1007/BF00326582

[7] May KJ, Whisson SC, Zwart RS, Searle IR, Irwin JA, et al (2002) Inheritance and mapping of 11 avirulence genes in Phytophthora sojae. Fungal Genet Biol 37: 1–12. 12223184 10.1016/s1087-1845(02)00027-0

[8] Tyler BM, Forster H, Coffey MD (1995) Inheritance of avirulence factors and restriction fragment length polymorphism markers in outcrosses of the oomycete *Phytophthora sojae*. Molecular Plant Microbe Interaction 8: 515–523.

[9] Chamnanpunt J, Shan WX, Tyler BM (2001) High frequency mitotic gene conversion in genetic hybrids of the oomycete Phytophthora sojae. Proc Natl Acad Sci U S A 98: 14530–14535. 11724938 10.1073/pnas.251464498 PMC64716

[10] Bhat RC, McBlain BA, Schmitthenner AF (1993) The inheritance of resistance to metalaxyl and to fluorophenylalaine in matings of homothallic Phytophthora sojae. Mycological research July 97: 865–870.

[11] Lamour KH, Mudge J, Gobena D, Hurtado-Gonzales OP, Schmutz J, et al (2012) Genome sequencing and mapping reveal loss of heterozygosity as a mechanism for rapid adaptation in the vegetable pathogen Phytophthora capsici. Molecular plant-microbe interactions: MPMI 25: 1350–1360. 10.1094/MPMI-02-12-0028-R 22712506 PMC3551261

[12] Vercauteren A, Boutet X, D’hondt L, Van Bockstaele E, Maes M, et al (2011) Aberrant genome size and instability of *Phytophthora ramorum* oospore progenies. Fungal genetics and biology: FG & B 48: 537–543. 21272658 10.1016/j.fgb.2011.01.008

[13] Hu J, Diao Y, Zhou Y, Lin D, Bi Y, et al (2013) Loss of heterozygosity drives clonal diversity of Phytophthora capsici in China. PLoS One 8: e82691 10.1371/journal.pone.0082691 24349339 PMC3861455

[14] Carter DA, Buck KW, Archer SA, Van der Lee T, Shattock RC, et al (1999) The detection of nonhybrid, trisomic, and triploid offspring in sexual progeny of a mating of Phytophthora infestans. Fungal Genet Biol 26: 198–208. 10361034 10.1006/fgbi.1999.1120

[15] MacGregor T, Bhattacharyya M, Tyler B, Bhat R, Schmitthenner AF, et al (2002) Genetic and physical mapping of Avrla in Phytophthora sojae. Genetics 160: 949–959. 11901113 10.1093/genetics/160.3.949 PMC1462023

[16] Gijzen M, Qutob D (2009) *Phytophthora sojae* and Soybean In: Lamour K, Kamoun S, editors. Oomycete Genetics and Genomics: Diversity, Interactions, and Research Tools: John Wiley & Sons, Inc. pp. 303–329.

[17] Tyler BM (2007) Phytophthora sojae: root rot pathogen of soybean and model oomycete. Molecular Plant Pathology 8: 1–8. 10.1111/j.1364-3703.2006.00373.x 20507474

[18] Dorrance A, Grünwald NJ (2009) *Phytophthora sojae*: Diversity among and within Populations In: Lamour K, Kamoun S, editors. Oomycete Genetics and Genomics: Diversity, Interactions, and Research Tools: John Wiley & Sons, Inc. pp. 197–212.

[19] Dorrance AE, Robertson AE, Cianzo S, Giesler LJ, Grau CR, et al (2009) Integrated Management Strategies for Phytophthora sojae Combining Host Resistance and Seed Treatments. Plant disease: an international journal of applied plant pathology 93: 875–882. 10.1094/PDIS-93-9-0875 30754536

[20] Qutob D, Tedman-Jones J, Dong S, Kuflu K, Pham H, et al (2009) Copy number variation and transcriptional polymorphisms of Phytophthora sojae RXLR effector genes Avr1a and Avr3a. PLoS ONE 4: e5066 10.1371/journal.pone.0005066 19343173 PMC2661136

[21] Shan WX, Cao M, Dan LU, Tyler BM (2004) The Avr1b locus of Phytophthora sojae encodes an elicitor and a regulator required for avirulence on soybean plants carrying resistance gene Rps1b. Molecular Plant-Microbe Interactions 17: 394–403. 15077672 10.1094/MPMI.2004.17.4.394

[22] Na R, Yu D, Chapman BP, Zhang Y, Kuflu K, et al (2014) Genome re-sequencing and functional analysis places the Phytophthora sojae avirulence genes Avr1c and Avr1a in a tandem repeat at a single locus. PLoS One 9: e89738 10.1371/journal.pone.0089738 24586999 PMC3933651

[23] Na R, Yu D, Qutob D, Zhao J, Gijzen M (2013) Deletion of the Phytophthora sojae avirulence gene Avr1d causes gain of virulence on Rps1d. Molecular plant-microbe interactions: MPMI 26: 969–976. 10.1094/MPMI-02-13-0036-R 23550527

[24] Yin W, Dong S, Zhai L, Lin Y, Zheng X, et al (2013) The Phytophthora sojae Avr1d gene encodes an RxLR-dEER effector with presence and absence polymorphisms among pathogen strains. Mol Plant Microbe Interact 26: 958–968. 10.1094/MPMI-02-13-0035-R 23594349

[25] Song T, Kale SD, Arredondo FD, Shen D, Su L, et al (2013) Two RxLR avirulence genes in Phytophthora sojae determine soybean Rps1k-mediated disease resistance. Molecular plant-microbe interactions: MPMI 26: 711–720. 10.1094/MPMI-12-12-0289-R 23530601

[26] Dong S, Yu D, Cui L, Qutob D, Tedman-Jones J, et al (2011) Sequence variants of the Phytophthora sojae RXLR effector Avr3a/5 are differentially recognized by Rps3a and Rps5 in soybean. PloS one 6: e20172 10.1371/journal.pone.0020172 21779316 PMC3136461

[27] Dong S, Yin W, Kong G, Yang X, Qutob D, et al (2011) Phytophthora sojae avirulence effector Avr3b is a secreted NADH and ADP-ribose pyrophosphorylase that modulates plant immunity. PLoS pathogens 7: e1002353 10.1371/journal.ppat.1002353 22102810 PMC3213090

[28] Dong S, Qutob D, Tedman-Jones J, Kuflu K, Wang Y, et al (2009) The Phytophthora sojae avirulence locus Avr3c encodes a multi-copy RXLR effector with sequence polymorphisms among pathogen strains. PLoS ONE 4: e5556 10.1371/journal.pone.0005556 19440541 PMC2678259

[29] Dou D, Kale SD, Liu T, Tang Q, Wang X, et al (2010) Different domains of Phytophthora sojae effector Avr4/6 are recognized by soybean resistance genes Rps4 and Rps6. Molecular plant-microbe interactions: MPMI 23: 425–435. 10.1094/MPMI-23-4-0425 20192830

[30] Qutob D, Chapman PB, Gijzen M (2013) Transgenerational gene silencing causes gain of virulence in a plant pathogen. Nature communications 4: 1349 10.1038/ncomms2354 23322037 PMC3562452

[31] Gijzen M, Ishmael C, Shrestha SD (2014) Epigenetic control of effectors in plant pathogens. Front Plant Sci 5: 638 10.3389/fpls.2014.00638 25429296 PMC4228847

[32] Kasuga T, Gijzen M (2013) Epigenetics and the evolution of virulence. Trends in microbiology 21: 575–582. 10.1016/j.tim.2013.09.003 24095304

[33] Long M, Keen NT, Ribeiro OK, Leary JV, Erwin DC, et al (1975) Phytophthora megasperma var. sojae: Development of wild-type strains for genetic research. Phytopathology 65: 592–597.

[34] Jablonka E, Raz G (2009) Transgenerational epigenetic inheritance: prevalence, mechanisms, and implications for the study of heredity and evolution. Q Rev Biol 84: 131–176. 19606595 10.1086/598822

[35] Rechavi O, Houri-Ze'evi L, Anava S, Goh WS, Kerk SY, et al (2014) Starvation-induced transgenerational inheritance of small RNAs in C. elegans. Cell 158: 277–287. 10.1016/j.cell.2014.06.020 25018105 PMC4377509

[36] Rechavi O, Minevich G, Hobert O (2011) Transgenerational inheritance of an acquired small RNA-based antiviral response in C. elegans. Cell 147: 1248–1256. 10.1016/j.cell.2011.10.042 22119442 PMC3250924

[37] Shao C, Li Q, Chen S, Zhang P, Lian J, et al (2014) Epigenetic modification and inheritance in sexual reversal of fish. Genome Res 24: 604–615. 10.1101/gr.162172.113 24487721 PMC3975060

[38] Iwasaki M, Paszkowski J (2014) Epigenetic memory in plants. EMBO J 33: 1987–1998. 10.15252/embj.201488883 25104823 PMC4195768

[39] Kelly WG (2014) Transgenerational epigenetics in the germline cycle of Caenorhabditis elegans. Epigenetics Chromatin 7: 6 10.1186/1756-8935-7-6 24678826 PMC3973826

[40] Forster H, Tyler BM, Coffey MD (1994) *Phytophthora sojae* races have arisen by clonal evolution and by rare outcrosses. Molecular Plant Microbe Interaction 7: 780–791.

[41] Dobrowolski MP, Tommerup IC, Blakeman HD, O'Brien PA (2002) Non-Mendelian inheritance revealed in a genetic analysis of sexual progeny of Phytophthora cinnamomi with microsatellite markers. Fungal Genet Biol 35: 197–212. 11929210 10.1006/fgbi.2001.1319

[42] Francis DM, Gehlen MF, St Clair DA (1994) Genetic variation in homothallic and hyphal swelling isolates of Pythium ultimum var. ultimum and P. utlimum var. sporangiferum. Molecular plant-microbe interactions: MPMI 7: 766–775. 7873781 10.1094/mpmi-7-0766

[43] Chandler V, Alleman M (2008) Paramutation: epigenetic instructions passed across generations. Genetics 178: 1839–1844. 18430919 10.1093/genetics/178.4.1839 PMC2323780

[44] Rutherford FS, Ward EWB, Buzzell RI (1985) Variation in virulence in successive single-zoospore propagations of *Phytophthora megasperma* f.sp. *glycinea*. Phytopathology 75: 371–374.

[45] Raffaele S, Farrer RA, Cano LM, Studholme DJ, MacLean D, et al (2010) Genome evolution following host jumps in the Irish potato famine pathogen lineage. Science (New York, NY) 330: 1540–1543. 10.1126/science.1193070 21148391

[46] Judelson HS, Tani S (2007) Transgene-induced silencing of the zoosporogenesis-specific NIFC gene cluster of Phytophthora infestans involves chromatin alterations. Eukaryotic cell 6: 1200–1209. 17483289 10.1128/EC.00311-06 PMC1951104

[47] Vetukuri RR, Asman AK, Tellgren-Roth C, Jahan SN, Reimegard J, et al (2012) Evidence for small RNAs homologous to effector-encoding genes and transposable elements in the oomycete Phytophthora infestans. PloS one 7: e51399 10.1371/journal.pone.0051399 23272103 PMC3522703

[48] Whisson S, Vetukuri R, Avrova A, Dixelius C (2012) Can silencing of transposons contribute to variation in effector gene expression in Phytophthora infestans? Mobile genetic elements 2: 110–114. 22934246 10.4161/mge.20265 PMC3429519

[49] Vetukuri RR, Asman AK, Jahan SN, Avrova AO, Whisson SC, et al (2013) Phenotypic diversification by gene silencing in Phytophthora plant pathogens. Commun Integr Biol 6: e25890 10.4161/cib.25890 24563702 PMC3917941

[50] Vetukuri RR, Avrova AO, Grenville-Briggs LJ, Van West P, Soderbom F, et al (2011) Evidence for involvement of Dicer-like, Argonaute and histone deacetylase proteins in gene silencing in Phytophthora infestans. Mol Plant Pathol 12: 772–785. 10.1111/j.1364-3703.2011.00710.x 21726377 PMC6640358

[51] Dorrance AE, Berry SA, Anderson TR, Meharg C (2008) Isolation, storage, pathotype characterization, and evaluation of resistance for *Phytophthora sojae* in soybean. Plant health progress: 10.1094/PHP-2008-0118-1001-DG

[52] Tyler BM, Tripathy S, Zhang XM, Dehal P, Jiang RHY, et al (2006) Phytophthora genome sequences uncover evolutionary origins and mechanisms of pathogenesis. Science 313: 1261–1266. 16946064 10.1126/science.1128796

[53] Martin FN, Bensasson D, Tyler BM, Boore JL (2007) Mitochondrial genome sequences and comparative genomics of Phytophthora ramorum and P. sojae. Current Genetics 51: 285–296. 17310332 10.1007/s00294-007-0121-6

