# Supplementary material for: Strain Specific Factors Control Effector Gene Silencing in Phytophthora sojae
Source: PLoS One. 2016 Mar 1;11(3):e0150530. doi: 10.1371/journal.pone.0150530 (PMC4773254; doi:10.1371/journal.pone.0150530)
Supplement: S2 Table — (DOCX) [file pone.0150530.s002.docx]

| **S2 Table. Virulence outcomes and *Avr3a* transcript detection in oospore progeny from self-fertilized *P. sojae* strain P6497** | | | | | | |
| --- | --- | --- | --- | --- | --- | --- |
| Progeny number | *Rps3a* (L83-570)^1^ | | *rps3a* (Williams)^1^ | | Fisher’s test *p*-value^2^ | *Avr3a* mRNA^3^ |
|  | Alive | Dead | Alive | Dead |  |  |
|  |  |  |  |  |  |  |
| 1 | 22 | 0 | 0 | 17 | 1.96E-11 | (+) |
| 2 | 22 | 0 | 0 | 17 | 1.96E-11 | (+) |
| 3 | 22 | 0 | 0 | 17 | 1.96E-11 | (+) |
| 4 | 22 | 0 | 0 | 17 | 1.96E-11 | (+) |
| 5 | 22 | 0 | 1 | 16 | 4.51E-10 | (+) |
| 6 | 22 | 0 | 3 | 14 | 4.51E-08 | (+) |
| 7 | 22 | 0 | 0 | 17 | 1.96E-11 | (+) |
| 8 | 22 | 0 | 0 | 17 | 1.96E-11 | (+) |
| 9 | 22 | 0 | 0 | 17 | 1.96E-11 | (+) |
| 10 | 22 | 0 | 0 | 17 | 1.96E-11 | (+) |
| 11 | 22 | 0 | 0 | 17 | 1.96E-11 | (+) |
| 12 | 22 | 0 | 0 | 17 | 1.96E-11 | (+) |
| 13 | 22 | 0 | 0 | 17 | 1.96E-11 | (+) |
| 14 | 22 | 0 | 0 | 17 | 1.96E-11 | (+) |
| 15 | 22 | 0 | 0 | 17 | 1.96E-11 | (+) |
| 16 | 22 | 0 | 0 | 17 | 1.96E-11 | (+) |
| 17 | 22 | 0 | 0 | 17 | 1.96E-11 | (+) |
| 18 | 22 | 0 | 0 | 17 | 1.96E-11 | (+) |
| 19 | 22 | 0 | 0 | 17 | 1.96E-11 | (+) |
| 20 | 22 | 0 | 0 | 17 | 1.96E-11 | (+) |
| 21 | 22 | 0 | 0 | 17 | 1.96E-11 | (+) |
| 22 | 22 | 0 | 0 | 17 | 1.96E-11 | (+) |
| 23 | 22 | 0 | 0 | 17 | 1.96E-11 | (+) |
| 24 | 22 | 0 | 0 | 17 | 1.96E-11 | (+) |
| 25 | 22 | 0 | 0 | 17 | 1.96E-11 | (+) |
| 26 | 22 | 0 | 0 | 17 | 1.96E-11 | (+) |
| 27 | 22 | 0 | 0 | 17 | 1.96E-11 | (+) |
| 28 | 22 | 0 | 0 | 17 | 1.96E-11 | (+) |
| 29 | 22 | 0 | 0 | 17 | 1.96E-11 | (+) |
| 30 | 22 | 0 | 0 | 17 | 1.96E-11 | (+) |
| 31 | 22 | 0 | 0 | 17 | 1.96E-11 | (+) |
| 32 | 22 | 0 | 0 | 17 | 1.96E-11 | (+) |
| 33 | 22 | 0 | 0 | 17 | 1.96E-11 | (+) |
| 34 | 22 | 0 | 0 | 17 | 1.96E-11 | (+) |
| 35 | 22 | 0 | 0 | 17 | 1.96E-11 | (+) |
| 36 | 22 | 0 | 0 | 17 | 1.96E-11 | (+) |
| 37 | 22 | 0 | 0 | 17 | 1.96E-11 | (+) |
| 38 | 22 | 0 | 0 | 17 | 1.96E-11 | (+) |
| 39 | 22 | 0 | 0 | 17 | 1.96E-11 | (+) |
| 40 | 22 | 0 | 0 | 17 | 1.96E-11 | (+) |
| 41 | 22 | 0 | 0 | 17 | 1.96E-11 | (+) |
| 42 | 22 | 0 | 0 | 17 | 1.96E-11 | (+) |
| 43 | 22 | 0 | 0 | 17 | 1.96E-11 | (+) |
| 45 | 22 | 0 | 0 | 17 | 1.96E-11 | (+) |
| 46 | 22 | 0 | 0 | 17 | 1.96E-11 | (+) |
| 47 | 22 | 0 | 0 | 17 | 1.96E-11 | (+) |
| 48 | 22 | 0 | 0 | 17 | 1.96E-11 | (+) |
| 49 | 22 | 0 | 0 | 17 | 1.96E-11 | (+) |
| 50 | 22 | 0 | 0 | 17 | 1.96E-11 | (+) |
| ^1^The number of plants for each condition tested varied due to seed limitation.  ^2^Probability value from Fisher’s exact test, on whether the kill rate on test plants (*Rps3a*) differs from the kill rate on control plants (*rps3a*)  ^3^Symbol (+) positive for *Avr3a* mRNA, (-) negative for *Avr3a* mRNA. All samples were positive for control *Actin* mRNA. | | | | | | |
