# Supplementary material for: Strain Specific Factors Control Effector Gene Silencing in Phytophthora sojae
Source: PLoS One. 2016 Mar 1;11(3):e0150530. doi: 10.1371/journal.pone.0150530 (PMC4773254; doi:10.1371/journal.pone.0150530)
Supplement: S3 Table — (DOCX) [file pone.0150530.s003.docx]

| **S3 Table. Small RNA sequencing of *P. sojae* strains and progeny** | | | | | | | |
| --- | --- | --- | --- | --- | --- | --- | --- |
| *P. sojae* strain or progeny^1^ | Virulence to *Rps3a*^2^ | *Avr3a* genotype^3^ | Biological replicate | Raw reads | Filtered reads^4^ | Matches *Avr3a*^5^ | Normalized matches^6^ |
| ACR10 | V | A/A | 1 | 7,947,184 | 5,814,735 | 247 | 42.48 |
| ACR10 | V | A/A | 2 | 16,737,749 | 14,164,977 | 190 | 13.41 |
| ACR10 | V | A/A | 3 | 55,253,829 | 12,371,012 | 180 | 14.55 |
| P6497 | A | A/A | 1 | 20,588,616 | 19,031,955 | 2 | 0.11 |
| P6497 | A | A/A | 2 | 51,664,873 | 16,130,242 | 1 | 0.06 |
| P6497 | A | A/A | 3 | 49,139,647 | 15,394,434 | 2 | 0.13 |
| P7076 | A | B/B | 1 | 4,640,922 | 7,355,632 | 0 | 0.00 |
| P7076 | A | B/B | 2 | 17,125,128 | 14,231,568 | 0 | 0.00 |
| F1_46_P6497 X ACR10 | A | A/A | 1 | 39,727,782 | 9,955,445 | 52 | 5.22 |
| F1_ 111_P6497 X ACR10 | A | A/A | 2 | 32,894,790 | 4,562,277 | 22 | 4.82 |
| F1_119_P6497 X ACR10 | A | A/A | 3 | 54,481,167 | 13,040,250 | 46 | 3.53 |
| F1_52_P6497 X ACR10 | V | A/A | 1 | 45,236,660 | 10,299,903 | 167 | 16.21 |
| F1_92_P6497 X ACR10 | V | A/A | 2 | 35,943,136 | 10,162,738 | 107 | 10.53 |
| F1_40_P6497 X ACR10 | V | A/A | 3 | 55,434,407 | 15,978,634 | 363 | 22.72 |
| F2_46-13_P6497 X ACR10 | A | A/A | 1 | 34,429,871 | 8,442,373 | 0 | 0.00 |
| F2_ 111-02_P6497 X ACR10 | A | A/A | 2 | 34,078,151 | 8,753,039 | 1 | 0.11 |
| F2_46-24_P6497 X ACR10 | A | A/A | 3 | 52,298,548 | 15,474,945 | 92 | 5.95 |
| F2_46-20 _P6497 X ACR10 | V | A/A | 1 | 39,226,667 | 12,357,396 | 290 | 23.47 |
| F2_111-04 _P6497 X ACR10 | V | A/A | 2 | 32,348,772 | 7,795,671 | 92 | 11.80 |
| F2_46-8 _P6497 X ACR10 | V | A/A | 3 | 40,779,796 | 12,943,151 | 251 | 19.39 |
| TC_64_P6497 X [F1_62(ACR10XP7076)] | A | A/A | 1 | 35,688,843 | 8,723,132 | 382 | 43.79 |
| TC_72_P6497 X [F1_62(ACR10XP7076)] | A | A/A | 2 | 32,459,629 | 6,986,718 | 38 | 5.44 |
| TC_52_P6497 X [F1_62(ACR10XP7076)] | A | A/A | 3 | 50,393,667 | 11,730,585 | 74 | 6.31 |
| TC_02_P6497 X [F1_62(ACR10XP7076)] | A | A/B | 1 | 33,727,991 | 8,650,732 | 0 | 0.00 |
| TC_31_P6497 X [F1_62(ACR10XP7076)] | A | A/B | 2 | 35,028,347 | 8,616,115 | 5 | 0.58 |
| TC_106_P6497 X [F1_62(ACR10XP7076)] | A | A/B | 3 | 49,023,913 | 12,202,826 | 34 | 2.79 |
| ^1^TC = test cross progeny from P6497 X [F_1__62(ACR10XP7076)], as numbered in Table 3  ^2^V = virulent; A = avirulent. Virulent strains and progeny are silenced for *Avr3a*, while avirulent strains and progeny express the gene.  ^3^*P. sojae* strains ACR10 and P6497 possess sequence identical *Avr3a* alleles but differ in expression  ^4^Number of sequences reads after trimming and filtering, according to procedures described in Material and Methods.  ^5^Number of reads that match the *Avr3a* gene, including the complete open reading frame and 100 bp flanking each end.  ^6^Number of matches normalized per million reads. | | | | | | | |
