# Supplementary material for: Strain Specific Factors Control Effector Gene Silencing in Phytophthora sojae
Source: PLoS One. 2016 Mar 1;11(3):e0150530. doi: 10.1371/journal.pone.0150530 (PMC4773254; doi:10.1371/journal.pone.0150530)
Supplement: S4 Table — (DOCX) [file pone.0150530.s004.docx]

| **S4 Table. Oligonucleotide primer sequences** | | | |
| --- | --- | --- | --- |
| Target gene/ marker | Primer name | 5’ to 3’ sequence | Applications |
| *Avr3a* | *Sp92-33F*  *Sp92-325R* | GCTGCTTCCTTCCTGGTT GC  GCTGCTGCCTTTTGCTTCTC | PCR of genomic DNA (Hybrid identification); RT-PCR transcript detection |
| *Avr3a* | *Avr3a-qRT-F*  *Avr3a-qRT-R* | TCGCTCAAGTTGTGGTCGTC  TCGACAGCGTCCTATCTTCG | Quantitative real time PCR |
| *Avh320* | *Avh320-87-F*  *Avh320-19-R* | AACGCTCTCGAAAGTGGC  AAAGAACTTCGACAG CC | PCR of genomic DNA (Hybrid identification) |
| *Scaf-29-M2* | *Scaf-29-M2-F*  *Scaf-29-M2-R* | CCCTCGAGAACGCCAACTT  CCTCGCTCGCCTTCATCC | PCR of genomic DNA (Hybrid identification) |
| *Actin* | *7-2g-MY (Actin A)-F*  *7-2g-MY-(Actin A)-R* | CGAAATTGTGCGCGACATCAAG  GGTACCGCCCGACAGCACGAT | RT-PCR and Quantitative real time PCR |
| Mitochondrial Sequence | *mt1-P5008-F*  *mt1-P5008-R* | TTTGGTGTATAGTTTCCCAACC  CGTGTTACTCACCCGTTCG | Identify maternal parent |
